# Familial Transmission of Human T-cell Lymphotrophic Virus: Silent Dissemination of an Emerging but Neglected Infection

**DOI:** 10.1371/journal.pntd.0002272

**Published:** 2013-06-13

**Authors:** Carlos Araujo da Costa, Karen Cristini Yumi Ogawa Furtado, Louise de Souza Canto Ferreira, Danilo de Souza Almeida, Alexandre da Costa Linhares, Ricardo Ishak, Antonio Carlos Rosário Vallinoto, José Alexandre Rodrigues de Lemos, Luisa Caricio Martins, Edna Aoba Yassui Ishikawa, Rita Catarina Medeiros de Sousa, Maísa Silva de Sousa

**Affiliations:** 1 Núcleo de Medicina Tropical, Universidade Federal do Pará, Belém, Pará, Brazil; 2 Section of Virology, Instituto Evandro Chagas, Ministério da Saúde, Ananindeua, Pará, Brazil; 3 Instituto de Ciências Biológicas, Universidade Federal do Pará, Belém, Pará, Brazil; George Mason University, United States of America

## Abstract

**Background:**

HTLV-1 is a retrovirus that causes lymphoproliferative disorders and inflammatory and degenerative diseases of the central nervous system in humans. The prevalence of this infection is high in parts of Brazil and there is a general lack of public health care programs. As a consequence, official data on the transmission routes of this virus are scarce.

**Objective:**

To demonstrate familial aggregation of HTLV infections in the metropolitan region of Belém, Pará, Brazil.

**Method:**

A cross-sectional study involving 85 HTLV carriers treated at an outpatient clinic and other family members. The subjects were tested by ELISA and molecular methods between February 2007 and December 2010.

**Results:**

The prevalence of HTLV was 43.5% (37/85) for families and 25.6% (58/227) for the family members tested (95% CI: 1.33 to 3.79, *P* = 0.0033). Sexual and vertical transmission was likely in 38.3% (23/60) and 20.4% (29/142) of pairs, respectively (95% CI: 1.25 to 4.69, *P* = 0.0130). Positivity was 51.3% (20/39) and 14.3% (3/21) in wives and husbands, respectively (95% CI: 0.04 to 0.63, *P* = 0.0057). By age group, seropositivity was 8.0% (7/88) in subjects <30 years of age and 36.7% (51/139) in those of over 30 years (95% CI: 0.06 to 0.34, *P*<0.0001). Positivity was 24.1% (7/29) in the children of patients infected with HTLV-2, as against only 5.8% (4/69) of those infected with HTLV-1 (95% CI: 0.05 to 0.72, *P* = 0.0143).

**Conclusion:**

The results of this study indicate the existence of familial aggregations of HTLV characterized by a higher prevalence of infection among wives and subjects older than 30 years. Horizontal transmission between spouses was more frequent than vertical transmission. The higher rate of infection in children of HTLV-2 carriers suggests an increase in the prevalence of this virus type in the metropolitan region of Belém.

## Author Summary

Human T-cell lymphotropic virus (HTLV) has a slow replication rate and infection is characterized by low morbidity and mortality, as well as silent transmission within the population. While rare, HTLV-associated diseases are usually debilitating and life-threatening. The virus is endemic in the state of Pará (Brazil), although there have been no studies of the distribution of the virus within the local population. The results of the present study confirm the existence of familial aggregations of HTLV infection in the metropolitan region of the state capital, Belém. Considerably higher rates of sexual transmission of HTLV from men to women were also demonstrated. Rates of infection were similar for the two virus types, although HTLV-2 appears to be increasing in the population. The frequency of positivity among family members increased in direct proportion to age and was associated with a relatively large proportion of asymptomatic carriers. In addition, widespread ignorance of the virus increases the risk of transmission. The available evidence indicates that significant human suffering is caused by this virus in patients suffering complications, and this is little justification for the lack of intervention on the part of public health authorities, which might impede the ongoing proliferation of this infection in the population.

## Introduction

The acronym HTLV is the internationally accepted abbreviation for human T-cell lymphotropic virus, which belongs to the family Retroviridae. This virus has been implicated as the etiological agent of lymphoproliferative disorders, inflammatory and degenerative diseases of the central nervous system, and some immune system disorders [1], [2]. The two types of this virus, HTLV-1 and HTLV-2, which were described in the early 1980s, share 66% of their genetic makeup and tropism in T cells. However, HTLV-1 preferentially infects CD4+ T lymphocytes, in a manner similar to the human immunodeficiency virus (HIV), whereas HTLV-2 preferentially infects CD8+ T lymphocytes [3]–[5].

About 20 million people are estimated to be infected with HTLV worldwide [6]. Infection patterns are heterogeneous, with distinct geographical clusters for the two subtypes, HTLV-1 and HTLV-2 [7]–[9]. While associations with a number of other health problems have been reported, the principal diseases related to infection with HTLV-1 include tropical spastic paraparesis or HTLV-1-associated myelopathy (TSP/HAM), progressive and disabling diseases of the nervous system [10], [11], adult T-cell leukemia/lymphoma, and malignant lymphoproliferative disease [12], [13].

Studies of HTLV transmission in endemic regions worldwide have indicated that the virus spreads silently, primarily from mother to child and through sexual contact [14]–[16]. In Brazil, reliable data on the transmission of HTLV are still limited due to the lack of a national infection control program [17]. In this case, studies of HTLV transmission routes are extremely important, especially given the prevalence of HTLV in some regions of the country. Both HTLV-1 and HTLV-2 are endemic in the Brazilian state of Pará [18]. The objective of the present study was to determine the seroprevalence of HTLV-1/HTLV-2 antibodies among relatives of confirmed HTLV carriers treated at the Tropical Medicine Center (NMT) in the state capital, Belém, with the subsequent molecular verification of positive cases, in order to confirm the existence of familial aggregation of HTLV infection in this metropolitan region.

## Materials and Methods

Between June, 2007, and December, 2010, blood samples were collected from relatives of the 85 HTLV outpatients being monitored at the clinic of the Infectious Diseases Service in Belém, Pará, Brazil, who agreed to participate in the study. All 85 of these cases had been diagnosed by detection of proviral HTLV-1 or HTLV-2 DNA and lived in the metropolitan region of Belém.

The inclusion of contact subjects (N = 227) was based on the criterion of family relationships, given known HTLV transmission routes. Given, samples were solicited from the spouse and mother of male subjects, and the mother, spouse and children of female subjects. Other family members were recruited based on the identification of HTLV-positive cases among the contacts already investigated, using the same selection criteria.

Each family member was tested for HTLV-1/HTLV-2 antibodies by ELISA (Ortho Diagnostic System, Inc., USA). All seropositive relatives were tested for Polymerase Chain Reaction/Restriction Fragment Length Polymorphism (PCR/RFLP) for the confirmation of infection and the identification of the viral genotype [19].

For analysis, the cases were divided into groups according to virus type (HTLV-1 and HTLV-2), symptoms (symptomatic or asymptomatic), and transmission route (vertical: mother to child; horizontal: between spouses). Cases with symptoms not related to diseases caused by HTLV and those with no or few symptoms were classified as asymptomatic. The symptomatic patients sought health care most frequently and most often presented symptoms consistent with injuries caused by HTLV, based on the criteria reported in the literature.

Variation related to gender, degree of kinship, virus type, and symptoms were evaluated statistically using Chi-square or Fisher's exact test, where appropriate, and Mann-Whitney's U. A 5% significance level was considered for all analyses. All the information collected during the interviews was stored in a DBMS MS Access 2007 database for processing and analysis. Access to this database was restricted to the authors of this study. All statistical analyses were run in the BioEstat 5.0 program [20].

This study complied with the principles outlined by the Declaration of Helsinki and was approved by the Human Research Ethics Committee of the Tropical Medicine Center of the Federal University of Pará. An informed consent form was signed by all the participants or their parents/legal guardians prior to the collection of samples and the clinical examinations.

## Results

Sixty-two (72.9%) of the 85 outpatients infected with HTLV at NMT were infected with HTLV-1 and 23 (27.1%) with HTLV-2. Forty-seven (55.3%) were female and 38 (44.7%) male. Mean age at the time of enrollment at the clinic was 43.1 years (SD ±12.9), ranging from 12 to 64 years. Most cases (76.5%) were aged between 30 and 60 years.

Overall, 37 (43.5%) of the HTLV outpatients were classified as symptomatic and 48 (56.5%) as asymptomatic. Symptoms were only seen in patients infected with HTLV-1 and included neurological signs in 21 subjects, cutaneous manifestations in 16, rheumatological symptoms in 14, and ocular manifestations in 11. Five patients were infected with *Strongyloides stercoralis* and some patients presented two or three symptoms.

A total of 227 relatives were screened for HTLV infection by serological examination, including 154 (67.8%) females and 73 (32.2%) males, with a mean age of 37.3 years (SD ±18.2), ranging from one to 83 years. Slightly more than half of these subjects (51.1%; 116/227) reported a family income equal to or less than the minimum wage, 41% (93/227) an income of two to five times the minimum wage, and the remaining 7.9% (18/227) an income of more than five times the minimum wage. Education levels were also distributed bimodally – while 48.9% (111/227) of the subjects were high school graduates, 30.4% (69/227) were illiterate. Of the remainder, 16.7% (38/227) had completed primary school and 4% (9/227) were college graduates.

Familial transmission of HTLV was observed in 37 of the 85 families investigated, of which, 43.5% had at least two infected members. Individual transmission was observed in 58 (25.6%) of the 227 subjects studied (95% CI: 1.33 to 3.79, *P = *0.0033).

**Figure 1:**
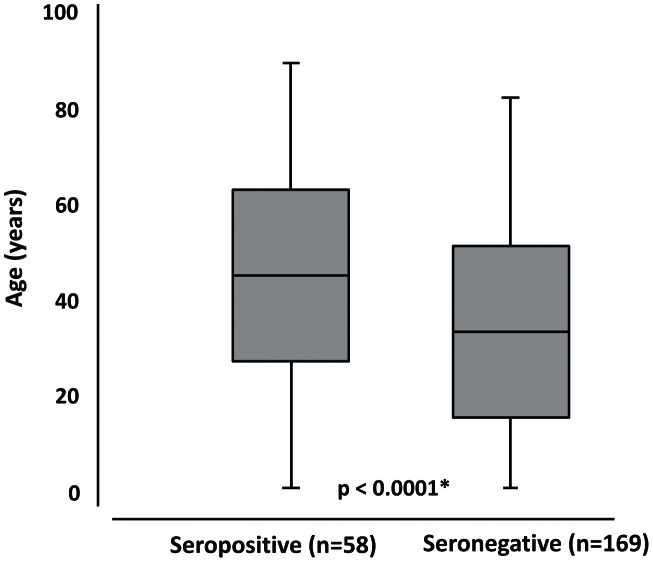
Box-plot comparing the age of the seropositive and seronegative relatives of HTLV-1/HTLV-2 carriers from Belém. Source: This image was made exclusively for the paper from data collected in Tropical Medicine Center, Belém, Pará, Brazil.* Chi-square test.

The ages of seropositive subjects ranged from 6 months to 90 years (mean ± SD = 45.4±17.8 years), whereas those of seronegative individuals were between 2 and 83 years, mean = 33.5±18.2 years (Figure 1). Seropositivity rates were 8.0% (7/88) in subjects of less than 30 years of age and 36.7% (51/139) in those of 30 years or older (Table 1). The mean age of HTLV-1 positive subjects was 46.5±18.2 years (range 6 months to 90 years), while that for HTLV-2 positive subjects was 43.1±17.1 years (range 17 to 78 years). This difference was not significant (*P* = 0.1588).

**Table 1:** Frequency of positive HTLV-1/HTLV-2 tests in relatives of HTLV carriers from Belém.

Relatives	Subjects studied N (%)	Seropositive N (%)	95% CI	*P*-value*
**Gender**			0.95 to 3.81	0.0932
female	154 (67.8)	45 (29.2)		
male	73 (32.2)	13 (17.8)		
**Age**			0.06 to 0.34	<0.0001
<30 years	88 (38.8)	7 (8.0)		
≥30 years	139 (61.2)	51 (36.7)		
**HTLV genotype**			0.48 to 1.79	0.9602
HTLV-1	163 (71.8)	41 (25.2)		
HTLV-2	64 (28.2)	17 (26.6)		
**Index cases**			0.68 to 2.26	0.5907
symptomatic	93 (41.0)	26 (28.0)		
asymptomatic	134 (59.0)	32 (23.9)		

Source: Tropical Medicine Center, Belém.

* Chi-square test.

Seroprevalence of HTLV was 29.2% (45/154) in female relatives versus 17.8% (13/73) in male relatives (Table 1). The analysis of infection rates by virus type indicated a seropositive rate of 25.2% (41/163) for HTLV-1 and 26.6% (17/64) for HTLV-2 (Table 1). The seropositivity rate was 28.0% (26/93) in relatives of symptomatic patients and 23.9% (32/134) in those of asymptomatic carriers (Table 1).

Vertical and sexual transmission occurred in 24 (28.2%) and 23 (27.1%) of the 85 families, respectively. Vertical transmission apparently occurred in 29 (20.4%) of 142 mother-son/daughter pairs, while sexual transmission probably occurred in 23 (38.3%) of 60 couples (95% CI: 1.25 to 4.69, *P = *0.0130).

There was no difference in the frequency of transmission from mother to daughter (20.0%, 18/90) or mother to son, 21.1% or 11/52 (Table 2). The mean duration of breast-feeding was 2 years and 3 months in seropositive relatives and 1 year and 7 months in seronegative one.

**Table 2:** Frequency of positive HTLV-1/HTLV-2 tests in mother-offspring and spouse-spouse dyads of patients from Belém.

Transmission	Subjects studied	Seropositive n (%)	95% CI	*P*-value
**Vertical**			0.46 to 2.49	0.9587*
daughters	90	18 (20.0)		
sons	52	11 (21.2)		
**Sexual**			0.04 to 0.63	0.0057**
wives	39	20 (51.3)		
husbands	21	3 (14.3)		

Source: Tropical Medicine Center, Belém.

* Chi-square test.

** Fisher's exact test.

The seroprevalence in children of patients infected with HTLV-2 and HTLV-1 was 24.1% (7/29) and 5.8% (4/69), respectively (95% CI: 0.05 to 0.72, *P = *0.0143). The seroprevalence rates observed in mothers of index cases were 46.4% (13/28) for HTLV-1 and 22.2% (2/9) for HTLV-2 (95% CI: 0.53 to 17.25, *P = *0.2616).

**Figure 2:**
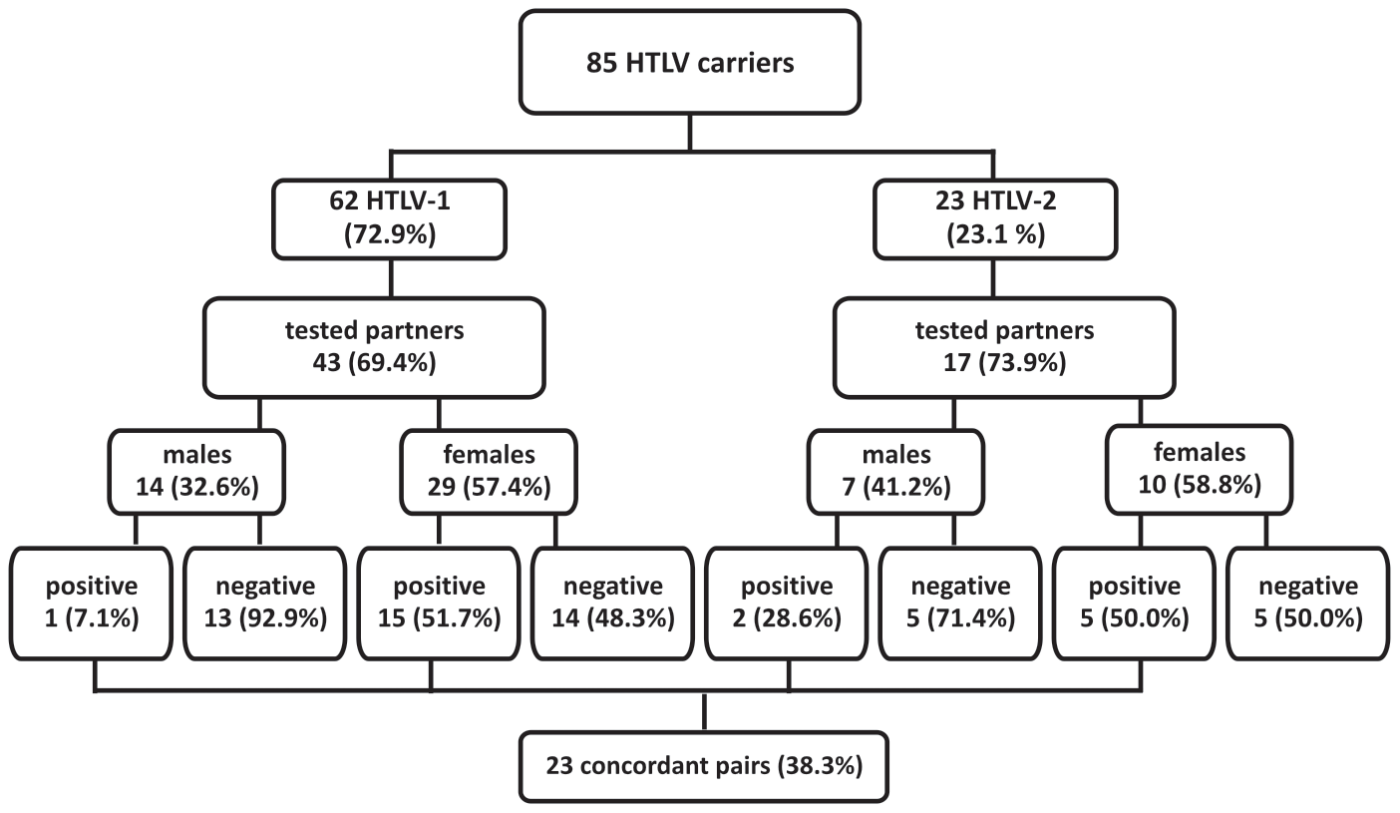
Schematic representation of the distribution of seropositive cases in the couples of families with HTLV-1/HTLV-2. Source: This image was made exclusively for the paper from data collected in Tropical Medicine Center, Belém, Pará, Brazil.

The prevalence of HTLV in wives (51.3%, 20/39) was significantly higher than that recorded in the husbands (14.3%, 3/21) of infected patients (Table 2). Similar rates were recorded for the wives of HTLV-1 patients (51.7%, 15/29) and those of HTLV-2 patients, with 50% or 5 of the 10 cases (Figure 2), although the difference was only significant in the former case (95% CI: 1.61 to 120.83, *P* = 0.0063), given the reduced sample size in the latter (95% CI: 0.55 to 11.41, *P* = 0.6221). All the couples reported maintaining a stable relationship. The average length of these relationships was 10.4 years in the cases where the spouses were seronegative, and 18.2 years in those in which they were seropositive.

**Figure 3:**
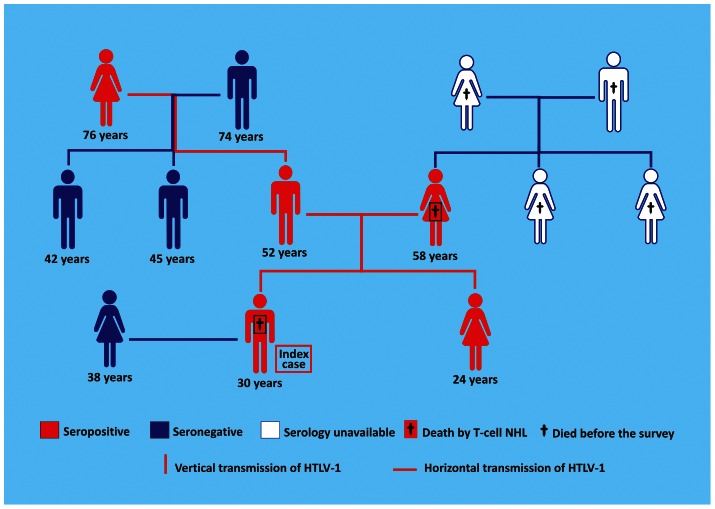
Heredogram showing the probable transmission routes of HTLV-1 in a family which suffered two deaths by T-cell non-Hodgkin lymphoma. Source: This image was made exclusively for the paper from data collected in Tropical Medicine Center, Belém, Pará, Brazil. NHL: Non-Hodgkin Lymphoma.

Familial transmission of HTLV-1 was observed in 27 of the 62 families investigated, of which, 43.5% had at least two infected members. Individual transmission was observed in 41 (25.2%) of the 163 subjects (95% CI: 0.2357 to 0.8051, *P = *0.0117). HTLV-1 infection was identified in five (55.56%) of nine members of a family in which there were three probable routes of vertical transmission and one probable route of sexual transmission, as well as two deaths by non-Hodgkin lymphoma (Figure 3).

## Discussion

The Tropical Medicine Center offers outpatient care to HTLV carriers resident in the state of Pará, in northern Brazil, which is located in the eastern extreme of the Amazon basin. More than 90% of the outpatients treated at this center are resident in the metropolitan region of the state capital, Belém. Up until the end of 2010, 177 outpatients infected with HTLV-1 and 72 infected with HTLV-2 (confirmed by the detection of proviral DNA) attended the center. The sample of 85 confirmed cases analyzed in the present study does not necessarily represent the prevalence of infection in the region, but reflects the reality of infection rates in the metropolitan region of Belém. Investigation of the transmission routes in these cases may nevertheless provide important insights into the transmission of HTLV in the region.

As HTLV has been shown to spread silently within families, the aim of the present study was to investigate familial aggregations of this infection based on the presence of the virus in at least two relatives of the outpatients. Familial aggregation was demonstrated by the significant difference in infection rates between carrier families (43.5%, 37/85) and the relatives tested (25.6%, 58/227), indicating that HTLV infection tends to occur in specific members of certain family groups. The seroprevalence rate of 25.6% recorded in family members in markedly higher than the mean rate for Brazilian blood donors in general (0.5%) or in the state of Pará in particular (0.91%) [18]. Slightly higher rates have been recorded in rural communities of northeastern Pará (1.14%) [19], but are still much lower than that recorded in the present study. This finding reinforces the need to investigate the proliferation of HTLV in the families of confirmed virus carriers.

A high prevalence of HTLV infection in the families of virus carriers has been demonstrated in a number of regions worldwide. In areas of high endemicity in Japan, the prevalence was 38.5% in the family members of infected individuals [21] and 47.7% in those of pregnant women with HTLV-1 [22]. A study conducted in Benin, West Africa, between 1991 and 1995 tested 138 relatives of 32 infected patients, and found a prevalence of 27.5%, a rate 18 times higher than the mean infection rate in the general population (1.5%). The male-to-female ratio was 1∶2 and the mean annual increase in new cases was 1.4% [23], [24]. Lu *et al*. (2001) [25] found a prevalence of 38.8% in 20 family members of HTLV-1-seropositive blood donors in Taiwan.

In a study conducted in the Brazilian state of Minas Gerais, involving family members of 352 HTLV-seropositive blood donors, the prevalence of HTLV-1 was 25.9% [15]. In the present study, the overall prevalence (25.6%), the prevalence rate in family members of HTLV-1 (25.2%), and the female-to-male ratio of approximately 2∶1 are consistent with the results with this study in Minas Gerais, and others in Brazil [15], [26].

Family members who were HTLV-positive were significantly older than those who were seronegative, which is consistent with previous studies, that have shown an increase in infection with age due to the eventual seroconversion of infections acquired early in life or the cumulative risk of infection through the course of an individual's life [27], [28]. It would therefore be informative to monitor the seronegative cases over the long term.

Cross-study comparisons of the prevalence rate of HTLV-2 recorded in family members in the present study (26.6%) were not possible due to the lack of data on this virus type. The HTLV-2 virus is endemic in eastern Amazonia, where the highest known rates of infection have been recorded (up to 40%), especially in indigenous communities [29], although the virus is also present in Belém, where the 2a subtype has been identified [30]. Molecular studies indicate that HTLV-2 is spreading from the indigenous communities to both rural and urban areas [31], although the presence of this virus is significantly lower in other regions of Brazil. Catalan-Soares *et al*. (2004) [15] reported a prevalence of only 2.5% (9/352) in Minas Gerais, for example. In the present study, the prevalence of HTLV-2 in all relatives screened by serology was 7.5% (17/227), i.e., three times higher than the general rate in Minas Gerais [15].

Little difference was found in the seropositivity rates of the relatives of symptomatic (28.0%) and asymptomatic (23.9%) outpatients, although the combined rate (25.6%) was 28 times higher than that recorded for blood donors from Pará (0.91%). In a Chilean study, an infection rate of 29.1% was recorded in the family members of carriers of TSP/HAM, a rate which was also 30 times higher than that recorded in the general population [32].

Positivity rates in mother-child pairs (20.4%) and children breast-fed for more than 6 months (34.0%) were similar to those recorded in previous studies. In Minas Gerais [15], the overall prevalence of HTLV infection in mother-child pairs was 20.9%. In a retrospective investigation of 120 Peruvian mothers infected with HTLV-1, the overall prevalence of infection was 23.3% (28/120) in children breast-fed for less than six months, and 32.6% (23/76) in those breast-fed for more than 6 months [33].

A number of studies have suggested that vertical transmission is more prevalent among women than men [15], [34]. In a recent study in Japan, however, the seroprevalence of HTLV in adolescents indicated that HTLV-1 infection is more common in males until the age of 20 years, after which sexual transmission from men to women becomes more likely, suggesting that transmission is more common from mother to son [35]. In the present study, the infection rates were similar in sons and daughters, while the number of positive cases increased significantly with age in children of both sexes.

The results of the present study revealed much higher rates of transmission to the offspring of HTLV-2 carriers when compared to HTLV-1 carriers (24.1% vs. 5.8%), although there are no data from other studies on which to base comparisons. Analysis of blood samples of native Kayapó from Kararaô village found specific reactivity to HTLV-2 in three of 26 subjects. Two samples were from a mother-child pair. Phylogenetic analysis of this pair showed high similarity (99.9%), thus providing molecular evidence of transmission of subtype HTLV-2c from a mother to her child [36].

Horizontal transmission between spouses was significantly more frequent than vertical transmission and was six times more likely to occur from men to women, than from women to men. As a consequence, infection tends to become more predominant in the women members of the family groups over time.

There is little consensus on the most common transmission route of HTLV. Vertical transmission was the principal route in a study in Nagasaki, Japan [14]. In a study of 82 relatives of 16 patients with HTLV-1 from Zaire, Central Africa, Liu et al. (1994) [37] recorded 15 seropositive mother-child pairs and concluded that vertical transmission is more prevalent. In the Brazilian study in Minas Gerais, however, horizontal transmission was the primary route of HTLV transmission [15].

With respect to the transmission of HTLV between spouses, a number of studies have shown that the virus is transmitted more efficiently from men to women. The results of an emblematic study conducted in the 1980s in Japan led to the projection that sexual transmission would be 60 times greater from men to women within 10 years [21]. The Miyazaki Cohort Study involving 534 couples found that global sexual transmission is independent of the woman's serological status, but rather depends on the duration of the relationship and on the husband's serological status [38]. This preferential male-to-female transmission of HTLV infection can be explained by biological factors [24] such as infected lymphocytes in the semen [39].

The analysis of risk factors through interviews with patients and relatives does not appear to be effective due to the subjectivity of self-reported data, given that patients may often be unsure of details or have biased memories. Most patients are already of advanced age, for example, and are unlikely to know whether they were breast-fed, and their mothers may have died or be unable to remember which of their children they breast-fed.

While the present study provides data on the prevalence of infection among family members, then, and has shown familial aggregations of HTLV infection, these data cannot be used to establish transmission routes reliably. However, further insights may be possible by the sequencing of genes to establish the identity of genomic chains between the members of pairs potentially involved in transmission.

Overall, the results of the present study are generally consistent with those published previously. Taken together, these studies indicate emphatically that HTLV is transmitted “silently” within family clusters. The investigation of HTLV infection in the families of virus carriers, whether symptomatic or asymptomatic, is essential in order to limit new infections and minimize the risks of serious diseases such as the two T-cell non-Hodgkin lymphoma cases observed in one of the families monitored in this study.

Interruption of the spread of the virus from these clusters will depend on the identification of cases in the population, although in Brazil, infection with HTLV is not treated as a public health problem and has been largely neglected [17], [40]. Despite this, HTLV is an example of the key neglected infectious diseases, which have been identified as priorities for research in Latin America and the Caribbean [41].

The present study confirms the existence of familial aggregations of HTLV infection in outpatients and, consequently, in the metropolitan region of Belém. Considering family relationships, the importance of sexual contact for the transmission of HTLV in the population, mainly from men to women, was demonstrated. Overall infection rates were similar among family members of carriers of HTLV-1 and HTLV-2. However, the higher rate of infection among children of HTLV-2 carriers compared to those of HTLV-1 carriers suggests an ongoing increase in the prevalence of HTLV-2 in the metropolitan region of Belém. The frequency of serological reactivity among family members increased in proportion to age and was associated with a large proportion of asymptomatic carriers. Overall, the general ignorance of the virus increases the risk of transmission considerably.

## Supporting Information

Checklist S1 **STROBE Checklist.**
(DOC)
